# Single-shot detection of 8 unique monochrome fringe patterns representing 4 distinct directions via multispectral fringe projection profilometry

**DOI:** 10.1038/s41598-021-88136-4

**Published:** 2021-05-14

**Authors:** Parsa Omidi, Mohamadreza Najiminaini, Mamadou Diop, Jeffrey J. L. Carson

**Affiliations:** 1 Imaging Program, Lawson Health Research Institute, St. Joseph’s Health Care, London, ON N6A 4V2 Canada; 2 School of Biomedical Engineering, Western University, London, ON N6A 3K7 Canada; 3 Spectral Devices Inc., 700 Collip Circle, Suite 125, London, ON N6G 4X8 Canada; 4 Department of Medical Biophysics, Western University, London, ON N6A 5C1 Canada; 5 Department of Surgery, Western University, London, ON N6A 5C1 Canada

**Keywords:** Imaging and sensing, Imaging techniques

## Abstract

Spatial resolution in three-dimensional fringe projection profilometry is determined in large part by the number and spacing of fringes projected onto an object. Due to the intensity-based nature of fringe projection profilometry, fringe patterns must be generated in succession, which is time-consuming. As a result, the surface features of highly dynamic objects are difficult to measure. Here, we introduce multispectral fringe projection profilometry, a novel method that utilizes multispectral illumination to project a multispectral fringe pattern onto an object combined with a multispectral camera to detect the deformation of the fringe patterns due to the object. The multispectral camera enables the detection of 8 unique monochrome fringe patterns representing 4 distinct directions in a single snapshot. Furthermore, for each direction, the camera detects two π-phase shifted fringe patterns. Each pair of fringe patterns can be differenced to generate a differential fringe pattern that corrects for illumination offsets and mitigates the effects of glare from highly reflective surfaces. The new multispectral method solves many practical problems related to conventional fringe projection profilometry and doubles the effective spatial resolution. The method is suitable for high-quality fast 3D profilometry at video frame rates.

## Introduction

Fringe projection profilometry (FPP) is a three-dimensional (3D) surface imaging technique that employs optical deflectometry to detect surface morphology of specular objects^1^. The technique has been successfully used in machine vision and medical imaging applications, where non-contact, full-field, and high-speed capabilities are needed. The FPP method projects fringe patterns on to an object and derives depth and height information from the phase distribution of the deformed fringes captured by one or more cameras. Generally, capturing more patterns leads to more accurate phase estimates, but the sequential projection of the patterns lengthens acquisition time and may not be suitable for highly dynamic measurements^2^. This situation has motivated many groups to develop methods to improve the speed of FPP through the use of fast switching digital projectors that cast fringes using monochromatic or RGB colored light^3–9^. To further enhance FPP speed, attempts have been made to generate multiple fringe patterns simultaneously. For example, groups have superimposed multiple fringe patterns each with a different spatial frequency^10,11^, a different color^12,13^, or different orientation^3,4,14–16^. Other groups have replaced the digital projector with faster devices. For example, Wakayama et al.^17^ used a fringe generation method that used three laser diodes and an electronically-controlled mirror to generate temporally phase-shifted fringe patterns at different wavelengths. Zhang et al. performed multiwavelength fringe illumination with eight different light-emitting diodes in combination with a multispectral camera^18^. The main advantage of this approach was that multiple fringe patterns were acquired in a single snapshot with each camera exposure, thereby overcoming the limitations of sequential pattern projection and imaging. However, the system was large and calibration was complex due to the large number of separate projection coordinates.

Phase distribution maps are computed from FPP data using a phase-demodulation algorithm. Algorithms can be categorized as phase-shifting (temporal phase stepping) and carrier-based (spatial phase stepping). The former requires at least three fringe patterns with known phase-step and generates accurate pixel-wise phase maps with minimal computational effort^19^. Phase maps retrieved from snapshots of fringe patterns can be computed with transform-based algorithms that utilize the Fourier transform (FT)^20^, windowed FT^3,21^, Wavelet transform^22^, or Hilbert transform^23^. Since these methods result in phase maps from individual camera frames, they are well-suited for dynamic 3D imaging; however, the methods have difficulty handling phase ambiguities. Phase ambiguities are dependant on many factors including low contrast fringe modulation, glare, shadows, stray light, noise, and surface discontinuities that cause phase shifts of more than 2π^24^. There have been attempts to mitigate the effects of phase ambiguities. Double-frame is one such method that uses a two-step phase shift approach^24–33^. Another method is to use aperiodic sinusoidal fringe patterns with cross-correlation between the measured intensity images^7,34^. The use of aperiodic fringe patterns solves the problem of 2π phase ambiguity for stereo based measurement systems but requires many images of unique fringe patterns.

Here, we report on a 3D FPP method that provides up to 8 unique fringe patterns during a single exposure. Our system employs a single light source equipped with a 4-band multispectral filter array (MFA) manufactured on a glass substrate. The MFA was comprised of four bandpass filters in a repeating mosaic arrangement with each filter 11 mm × 11 mm in size. With the MFA in the illumination path, a multispectral structured light pattern was generated on the object. We then utilized a multispectral camera to differentiate the light patterns at each band. After multispectral image processing, unique fringe patterns oriented every 45° with two complementary fringe patterns π-phase shifted with respect to each other at each orientation were recovered. The method provided the necessary object data to take advantage of robust reconstruction algorithms that implement π-phase shifted fringe patterns, but with the advantage of acquiring the data in a single camera exposure with a single light source from a single projection coordinate. Below, we introduce the multispectral fringe pattern profilometry (MFPP) method and provide the first performance tests.

## Results

**Figure 1:**
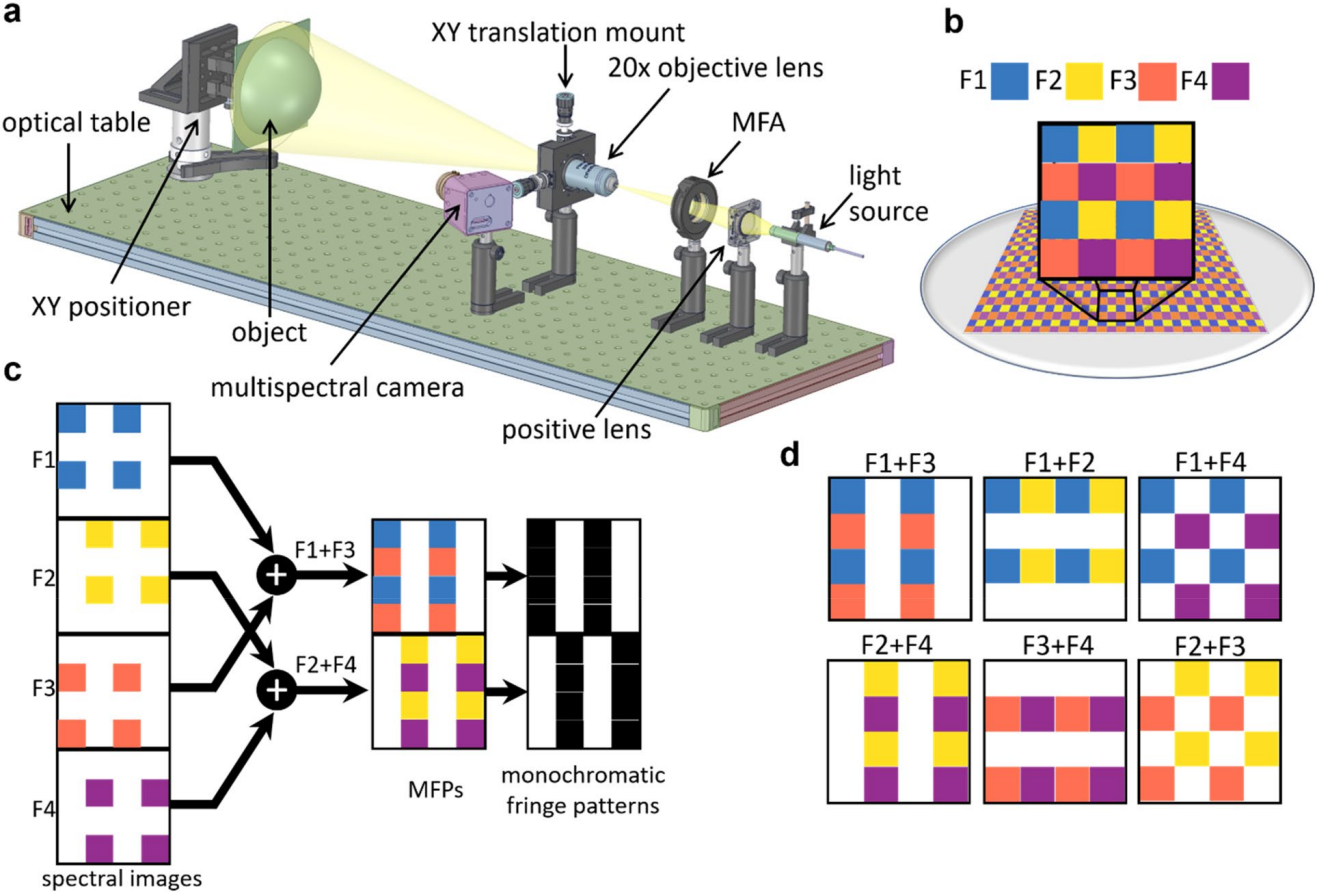
(**a**) Schematic illustration of the setup consisting of a halogen light source, multispectral filter array (MFA), focussing lens, object, and snapshot multispectral camera. (**b**) The MFA consisted of a 2D array of four distinct filters at different wavelengths: 580 nm, 660 nm, 735 nm, and 820 nm indicated by blue (F1), yellow (F2), orange (F3), and purple (F4), respectively. (**c**) Schematic of 2 vertical MFPs generated by combining pairs of spectral images of the object to form 2 unique monochromatic fringe patterns. For clarity, a single 4 × 4 pixel area is shown for each filter type, but the concept extends across all pixel data in each spectral image. (**d**) Schematic of all 6 MFPs, distinguishable as 8 unique monochromatic fringe patterns.

A setup of the apparatus is shown in Fig. 1a. Light from a halogen lamp was focussed onto a MFA (Spectral Devices Inc., London, Canada). The MFA was comprised of a 2 × 2 arrangement of square bandpass filters that was repeated in a 2D Bayer-like pattern^35,36^. The filters had peak optical transmission at 580 nm (F1), 660 nm (F2), 735 nm (F3), and 820 nm (F4) (Fig. 1b). An image of the MFA was focussed onto the target using a 20 × microscope objective resulting in a multispectral dot pattern that completely covered the target. An image of the pattern on the target was acquired with a snapshot multispectral camera with identical spectral response characteristics to the MFA (MSC-AGRI-1-A, Spectral Devices Inc., London, Canada^37^). With this setup, combinations of spectral dot patterns generated 6 distinct multispectral fringe patterns (MFP) directed in the vertical, horizontal, and diagonal directions (Fig. 1c,d). From these 6 MFPs, eight monochrome fringe patterns were extracted using a fringe extraction algorithm based on the Fourier transform^11^. The algorithm was capable of reliably extracting vertical, horizontal, and diagonal monochromatic fringe patterns from the vertical, horizontal, and diagonal MFPs, respectively.

**Figure 2:**
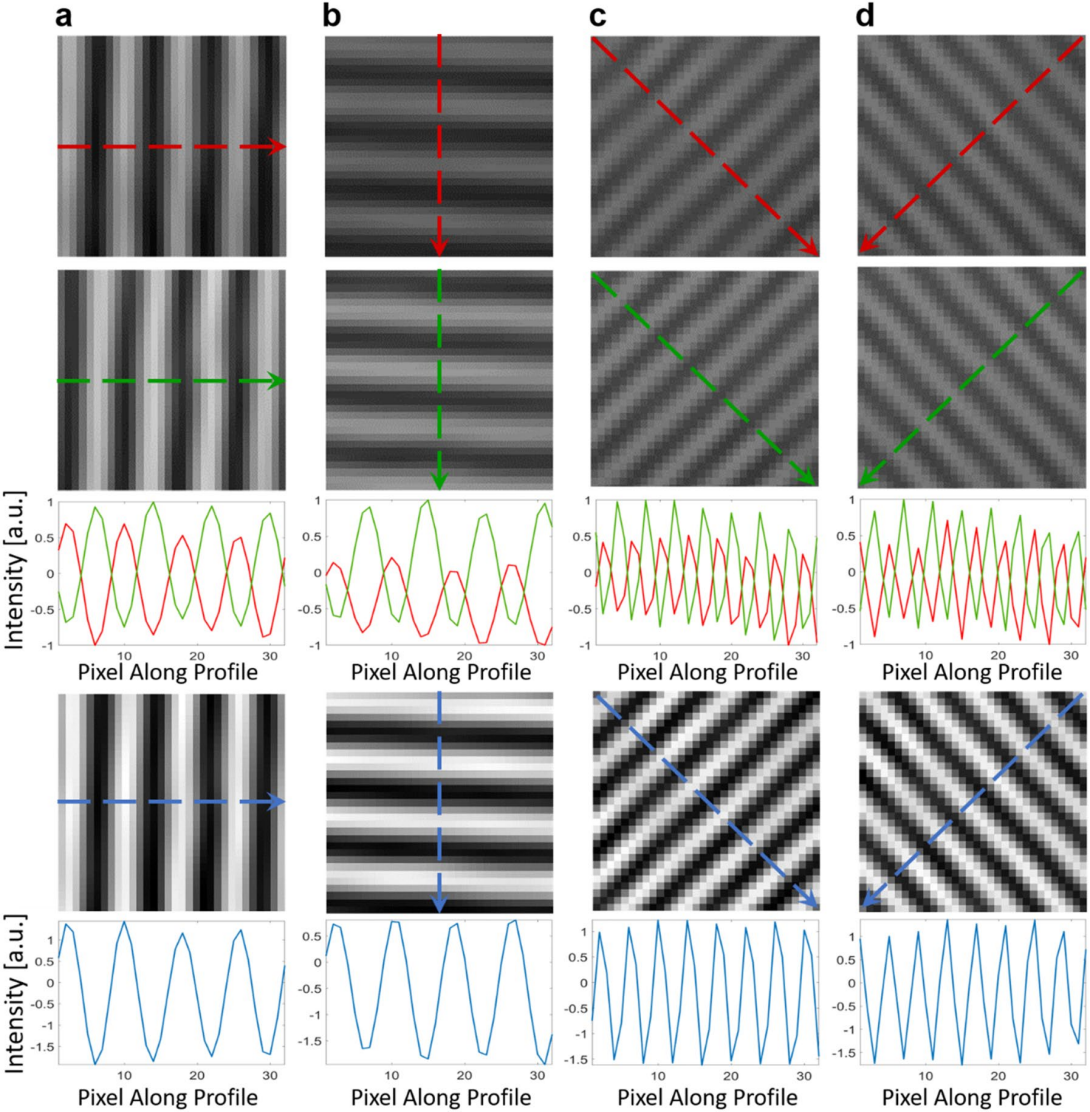
Subtraction of the complementary fringe patterns to produce a background-corrected and noise-reduced fringe pattern. (**a**) Images of a vertical fringe pattern, (**b**) horizontal fringe pattern, (**c**) 45-degree fringe pattern, and (**d**) 135-degree fringe pattern relative to the x-axis. From top to bottom: Images of the first complementary fringe pattern, second complementary π-phase shifted fringe pattern, color-coded intensity line profiles for the corresponding dashed lines indicated in the complementary fringe patterns, the DFP resulting from the subtraction of the first complementary fringe pattern from the second complementary fringe pattern, and the line profile for the corresponding dashed lines indicated in the DFP.

Furthermore, for each direction, the pair of complementary fringe patterns were π-phase shifted with respect to each other. Subtraction of one fringe pattern from its complement resulted in a differential fringe pattern (DFP) that was corrected for DC offset and had enhanced contrast (Fig. 2). Removal of the DC offset is known to improve the phase-demodulation performance of the FT algortihm^38^. In the FT phase-demodulation algorithm, the fringe pattern was first converted to the frequency domain, which provided two fundamental components due to the periodicity of the sinusoidal fringes. One fundamental component was selected and transferred back to the spatial domain. The lack of a DC offset, thanks to this filter selection process, enabled a wider bandwidth that extended toward lower frequencies without introducing leakage of the DC component into the chosen fundamental component. The wider bandwidth filter resulted in enhanced resolution during phase-demodulation and enabled lower frequency fringes to contribute to the extracted phase data. Sensitivity to lower fringe frequencies also provided the opportunity to image more distant targets, since the divergence of the illumination pattern resulted in larger fringes as the illuminator to object distance increased. Figure 2 shows an example of 4 pairs of complementary fringe patterns obtained within a single camera exposure of a rigid flat plate as the object. The intensity line profiles clearly show the π-phase shift between the complementary fringe patterns. Processing of the complementary fringe patterns resulted in differential fringe patterns with lower background and higher contrast.

System performance was examined through a series of experiments with complex test objects and reconstruction with the FT phase-demodulation algorithm. In our system configuration, the vertical direction was perpendicular to the projector-camera baseline and was the most sensitive fringe pattern direction^39^. However, the use of additional cameras oriented around the projector would give additional sensitivity to fringes perpendicular to each projector-camera baseline providing a best-case scenario for making use of all of the fringe patterns.

**Figure 3:**
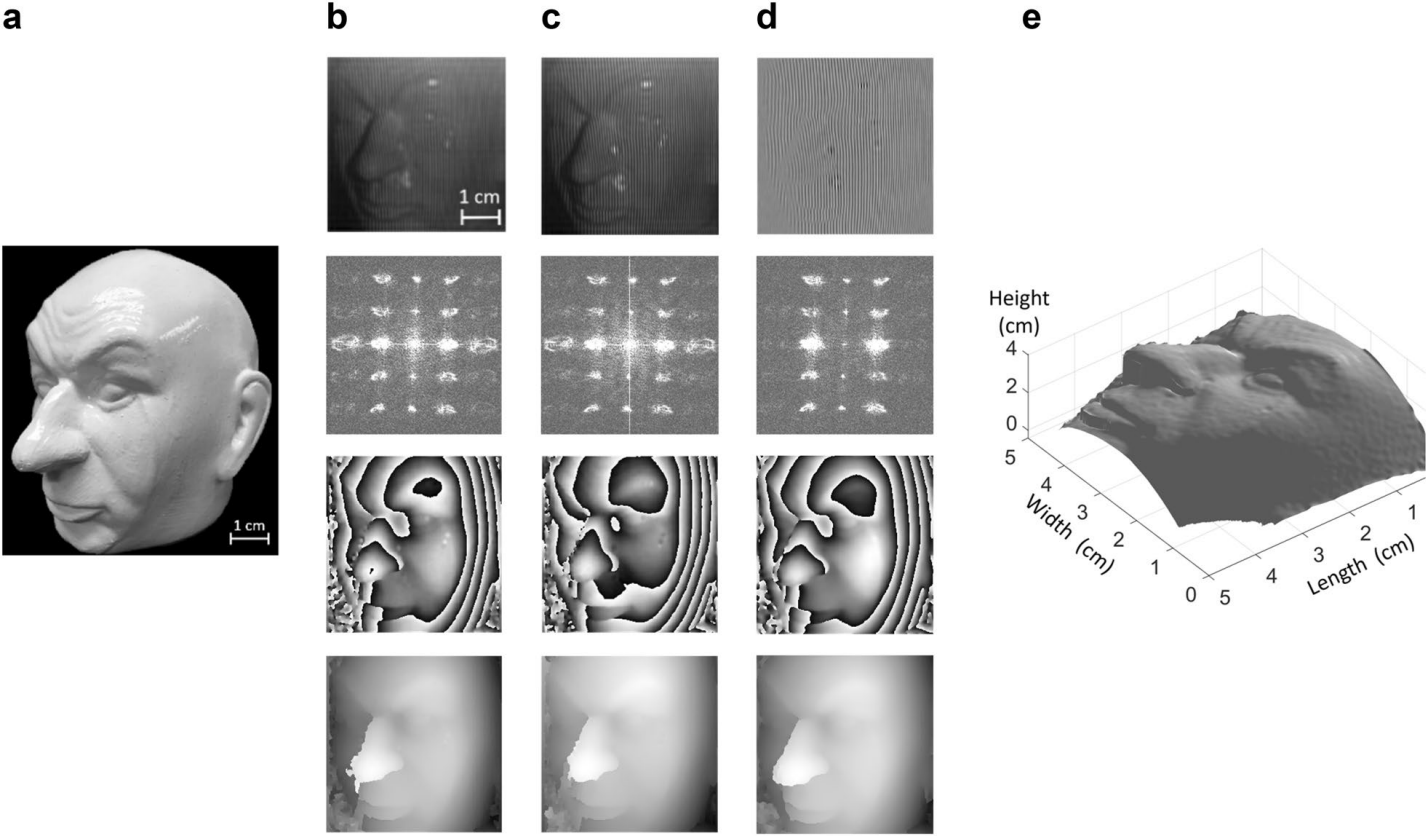
Phase demodulation for a head phantom. (**a**) Photograph of the head phantom. (**b**) Vertical fringe pattern acquired with MFPP method (top), the 2D frequency spectrum of the fringe pattern (second from top), wrapped phase computed from 2D frequency spectrum (second from bottom), and unwrapped phase (bottom) computed from the wrapped phase. (**c**) Similar to (**b**), except results are for the vertical π-phase shifted fringe pattern complementary to (**b**). (**d**) Similar to (**b**), except results are for the differential fringe pattern computed by subtracting the fringe pattern in **c** from the fringe pattern in (**b**). (**e**) 3D visualization of the object computed from the unwrapped phase in (**d**) after applying phase to height conversion.

Figure 3 shows results from an experiment with the MFPP system using a head phantom. A photograph of the head phantom is shown in Fig. 3a. Figure 3b shows one complementary vertical fringe pattern obtained from the head phantom, the frequency spectrum computed from the fringe pattern, the resulting wrapped phase, and the resulting unwrapped phase from top to bottom, respectively. The results for the complementary π-phase shifted vertical fringe pattern are shown in Fig. 3c and presented in a similar order to Fig. 3b. Similarly, the results for the DFP computed on the complementary vertical fringe patterns in Fig. 3b,c are shown in Fig. 3d. From the frequency spectra, it was apparent that the background illumination, present as low-frequency components near the center of the 2D spectra, was diminished after subtraction of the complementary fringe pair. Furthermore, glare was greatly reduced. For example, the bright features above the eye and below the nose in the top row of Fig. 3b,c were not as evident in the DFP (Fig. 3d top row). The glare was unavoidable since a directional illuminator was used for fringe projection, which is prone to generating specular reflections. Since the two complementary patterns result in similar specular reflections, the resulting glare patterns detected by the multispectral camera were nearly identical, thereby enabling glare removal by simple subtraction. Figure 3e shows the 3D visualization of the head phantom after applying a non-linear phase to height conversion to the unwrapped phase obtained from the subtracted pattern.

**Figure 4:**
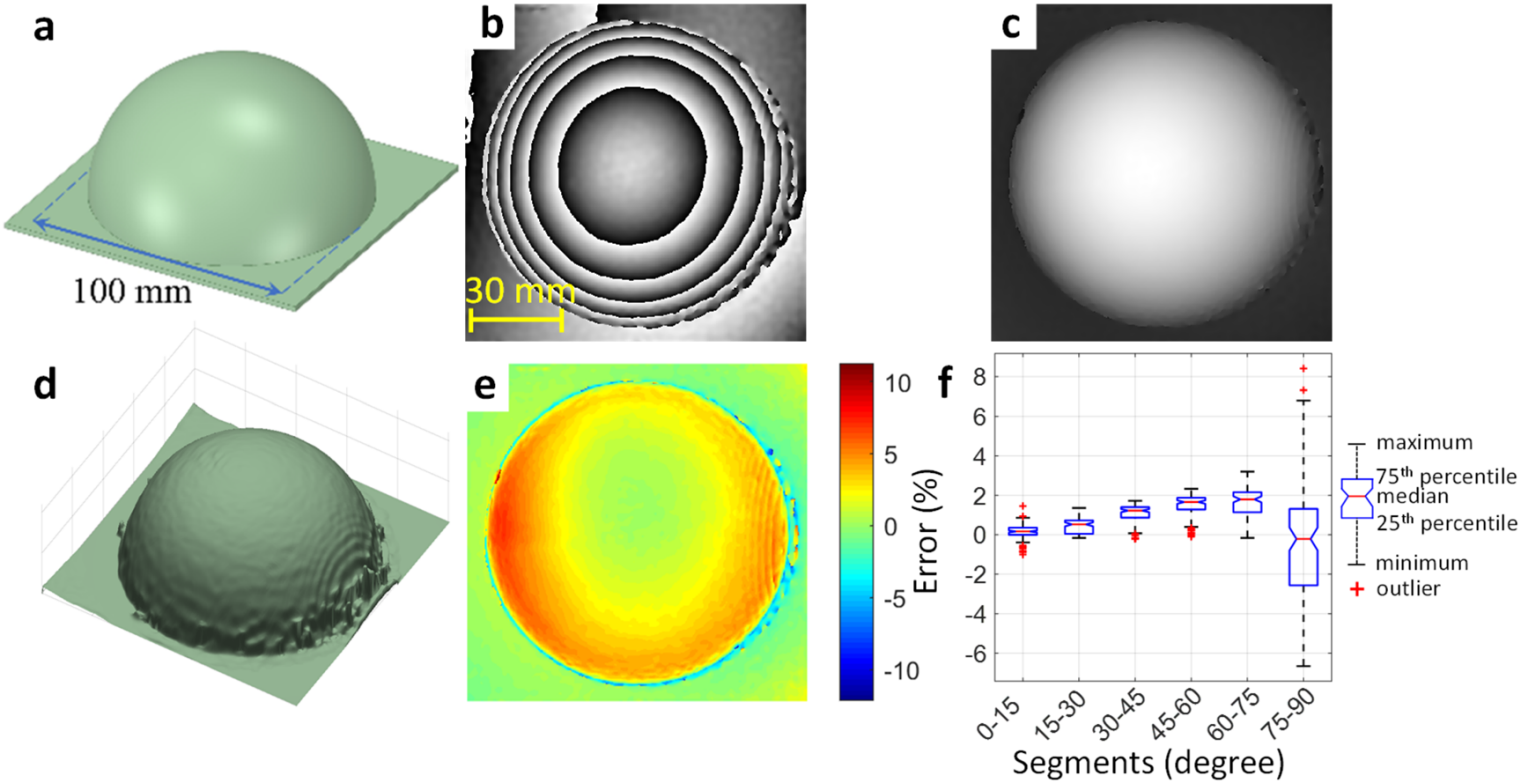
Accuracy test of MFPP. (**a**) CAD model of the target consisting of a hemisphere with 100 mm diameter. (**b**), (**c**) Wrapped and unwrapped phase maps, respectively. (**d**) 3D representation of the unwrapped phase after phase-to-height conversion. (**e**) Color-coded error map between the measured and expected height. (**f**) Statistical error in relation to the orientation of the surface normal toward the MFPP optical axis.

**Figure 5:**
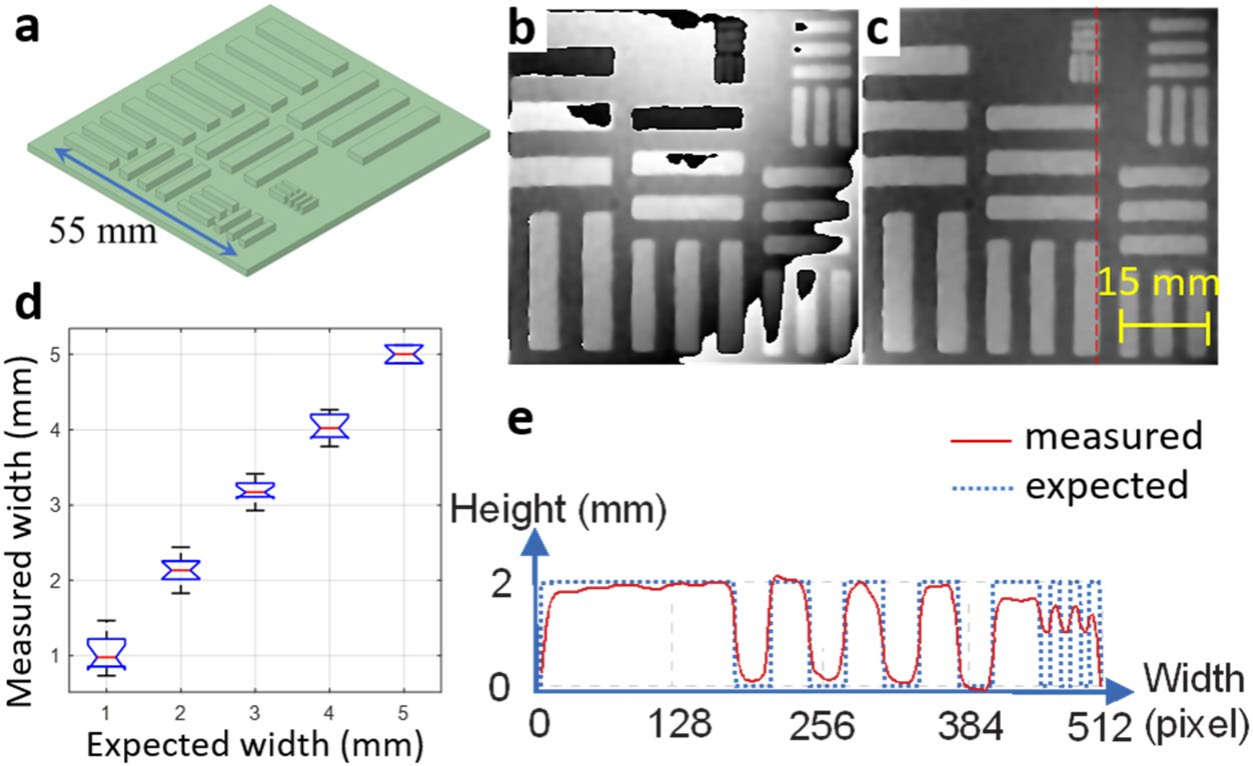
Lateral resolution test of MFPP. (**a**) CAD model of the target consisting of five groups of bars of varying width (1–5 mm in steps of 1 mm). (**b**), (**c**) Wrapped and unwrapped phase maps, respectively. (**d**) FWHM of the measured width against the expected width of the bars for different groups. (**e**) Profile of the measured height corresponding to the red dashed line in panel (**c**).

**Figure 6:**
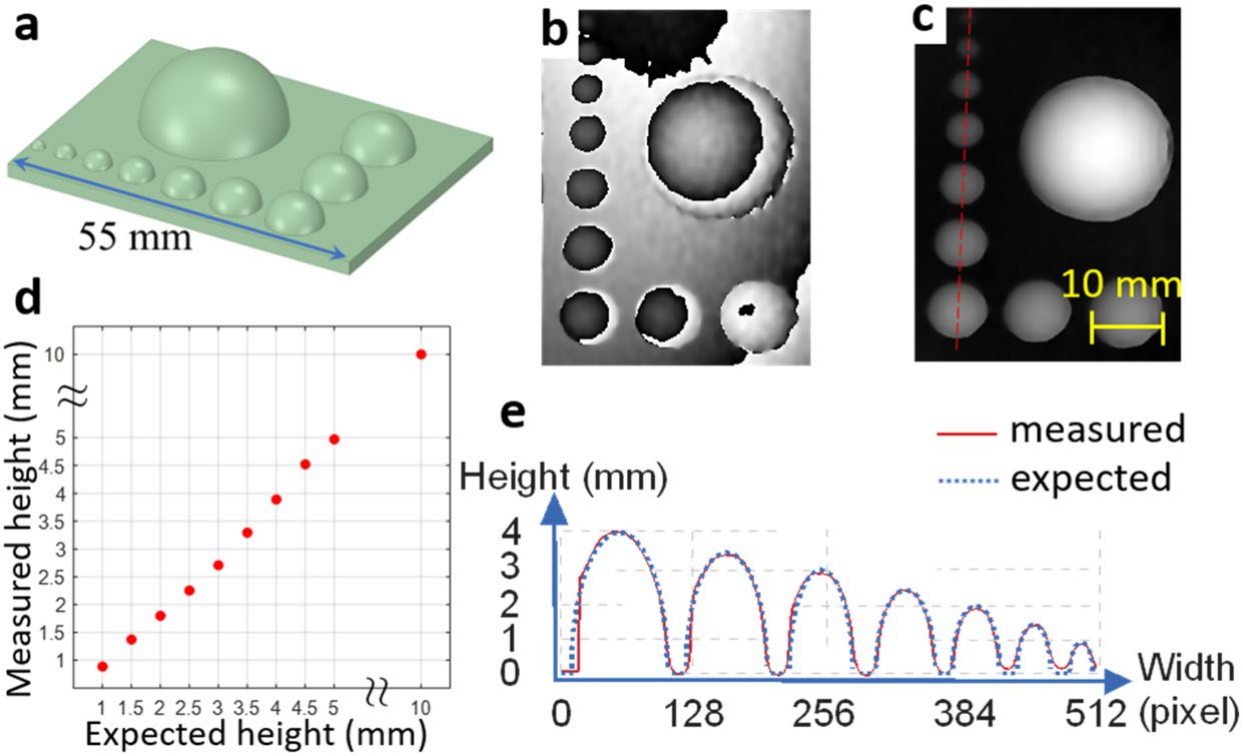
Axial resolution test. (**a**) CAD model of the axial resolution target consisting of ten hemispheres of different radius (10, 5, 4.5, 4, 3.5, 3, 2.5, 2, 1.5, and 1 mm). (**b**), (**c**) Wrapped and unwrapped phase maps, respectively. (**d**) Measured heights of the hemispheres against the expected values. (**e**) Measured height corresponding to the red dashed line in (**c**).

Figures 4, 5, and 6 show the accuracy, lateral resolution, and axial resolution tests of the MFPP system that were acquired using a set of stationary targets. The targets were 3D printed with PLA and had a dimensional accuracy of 0.2 mm. Figure 4 contains a hemispherical target with a diameter of 100 mm (3D model shown in Fig. 4a). The target represents a smooth object without any sudden changes to avoid potential phase artifacts due to the Fourier phase demodulation algorithm. Using MFPP, the wrapped and unwrapped phase estimates (Fig. 4b,c, respectively) were obtained using a single 10 ms camera exposure. The overall shape of the hemisphere was reproduced accurately, except near the base which was susceptible to phase ambiguity due to shadowing. Also, the bandpass filter used in FT removed higher frequencies in the Fourier domain which led to a loss of sharpness at the edges. Moreover, the vertical fringe pattern under-sampled the left and right sides of the hemisphere and resulted in degradation of the phase maps in these areas; yet the largest error was ~ 10%. A 3D visualization of the unwrapped phase map is shown in Fig. 4d. Figure 4e shows a color-coded error map where the measured height was compared with the expected height. Figure 4f represents the statistical result of the error map. In this representation, the image was segmented into six different regions based on the orientation of the surface normal toward the optical axis of the illuminator and the camera. The results of the lateral resolution are shown in Fig. 5. The target is shown in Fig. 5a and had five different groups of bars; each group consisted of six bars. Across the groups, the bar width varied from 1 to 5 mm in steps of 1 mm, the bar length was five times the bar width, and all bars had a height of 2 mm. Figure 5b,c show the estimated wrapped and unwrapped phase, respectively. In Fig. 5d, the full width at half maximum (FWHM) of the measured widths of the bars for different groups are plotted against the expected values. Figure 5e shows a line profile that passes through the smallest group and corresponds to the red dashed line in Fig. 5c. The system could accurately detect the phase changes for elements with a width of 2 mm or greater.

The target represented in Fig. 6a was designed to measure the axial resolution of the system and contained ten hemispheres of different radius from 1 to 10 mm. Similar to the results shown in Fig. 4, shadowing and under-sampling was evident in the wrapped and unwrapped phase maps of the hemispheres (Fig. 6b,c). A plot of the measured height of the hemispheres vs the expected height is represented in Fig. 6d. The line profile shown in Fig. 6e passes through the center of the seven smallest hemispheres. The smallest hemisphere in the target had a 1 mm height, which was correctly estimated by the system. Since a 20× objective lens was used with a pupil-object distance of 60 cm, the dot size on the surface of the object was 1 mm and only two dots covered the smallest hemisphere.

We next tested the ability of MFPP to capture dynamic 3D surface maps by mounting an object on the platform of a rotation stage and spinning the stage platform at 10 RPM. The reflected MFPs were captured from the object at 30 FPS during one 360° rotation giving rise to 180 frames for analysis.

**Figure 7:**
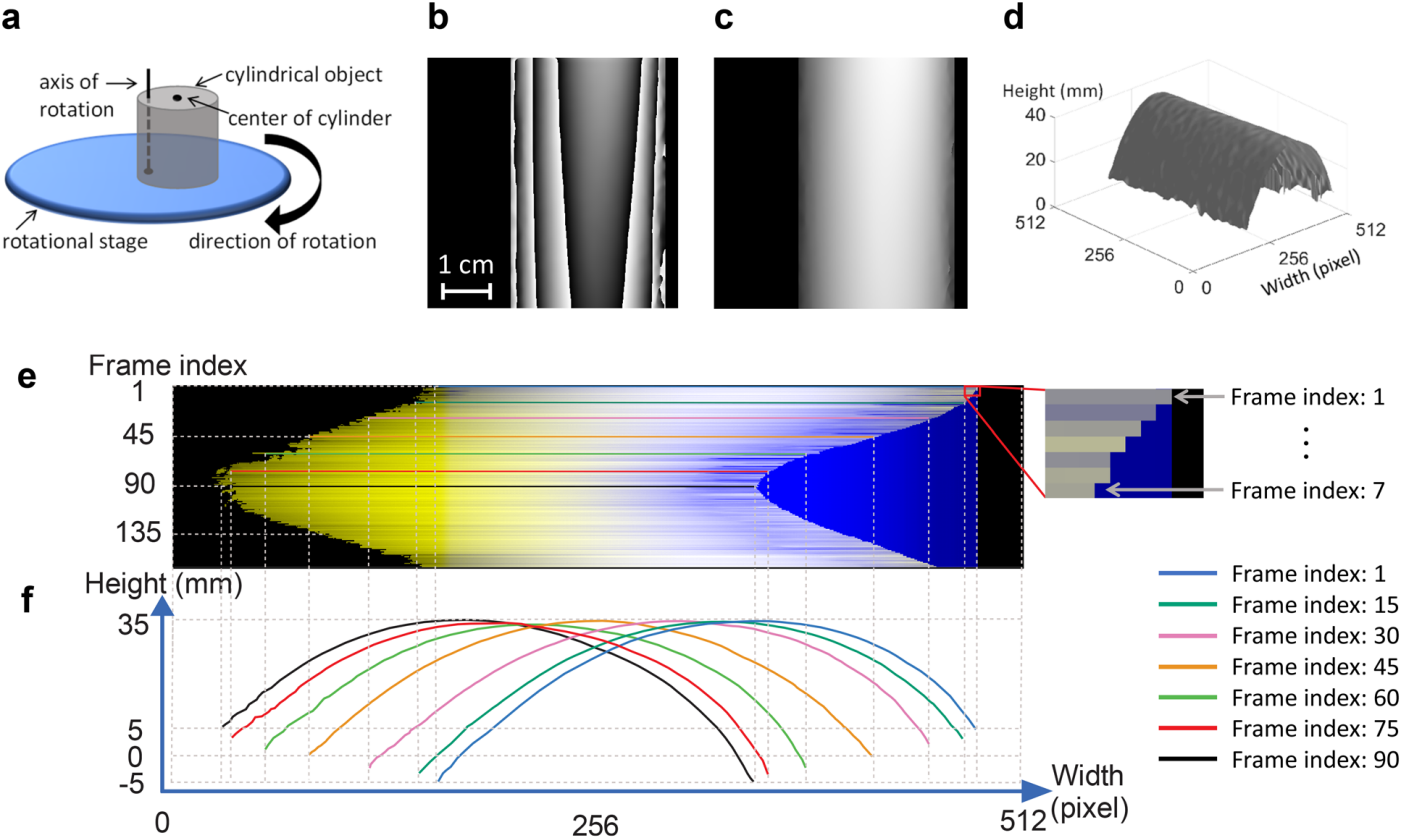
Analysis of motion artifacts by scanning a rotating object. (**a**) Reflective cylindrical object with 35 mm radius mounted on a rotational stage. The center of the cylinder was located 20 mm from the center of the stage. (**b**–**d**) Wrapped phase map, unwrapped phase map, and the 3D plot of the unwrapped phase map for frame index 1 (when the cylinder was located on the right side of the stage from the perspective of the camera). (**e**) Color-coded line profiles for continuous frame indices from 1 to 160. The blue area shows the first frame index, and the yellow lines show the line profiles for all indexes. The magnified area (right panel in **e**) shows partial frames 1–7 when the cylinder moved from right to left as the stage rotated. (**f**) Color-coded height profiles of the cylinder corresponding to the select frame indices indicated in (**e**).

Figure 7 shows an experiment with a rigid cylinder (radius = 35 mm) that was placed on the rotation stage approximately 20 mm from the axis of rotation (Fig. 7a). This setup was designed to estimate motion artifacts that might arise due to perspective errors. In principle, reconstruction of each snapshot should result in a cylinder, even though the location of the object in the field of view moves with each frame. Figures 7b,c,d show the wrapped phase map, unwrapped phase map, and 3D plot of the surface from the first acquired frame, respectively. The stack of line profiles acquired from each frame clearly shows the sinusoidal movement of the object, which is expected due to rotation at constant angular velocity. The left side of the line profiles is noisier likely due to shadowing resulting from the fact that the illuminator was located on the right side of the cylinder. Figure 7f shows selected height profiles for the corresponding frames indicated in Fig. 7e**.** As the cylinder was rotated, the left side of the cylinder became more visible as the lateral position of the cylinder became more central to the optical axis of the camera.

**Figure 8:**
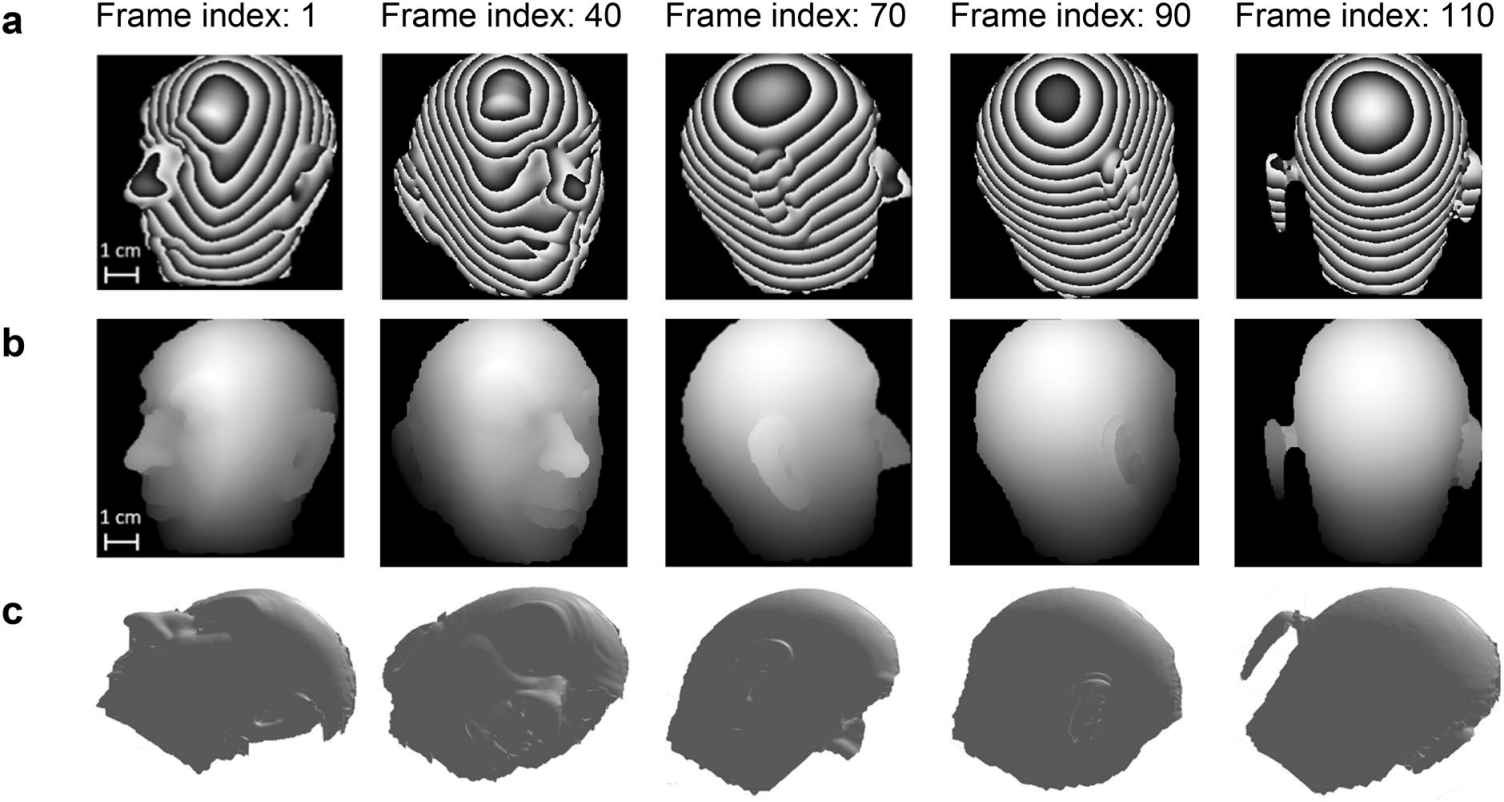
Dynamic 3D surface scanning of a rotating object. (**a**) Wrapped and (**b**) unwrapped phase maps for five selected frames of a rotating head phantom at different stages of object’s rotation. (**c**) 3D visualization of the unwrapped phase maps.

The next experiment shows a quality measurement where the stage spun the head phantom about its central inferior-superior axis. Figures 8a,b show the wrapped and unwrapped phase for five frames captured at different stages during the rotation. Figure 8c shows a 3D visualization of the unwrapped phase maps. The system could easily track the object and was unaffected by motion artifacts. However, the results show some artifacts caused by poor illumination in high slope areas (e.g., the edges of the head) and shadowing (e.g., the ear in the frame index 110). Nevertheless, these results clearly illustrate the snapshot capability of MFPP and demonstrate its ability to provide video rate 3D surface mapping.

**Figure 9:**
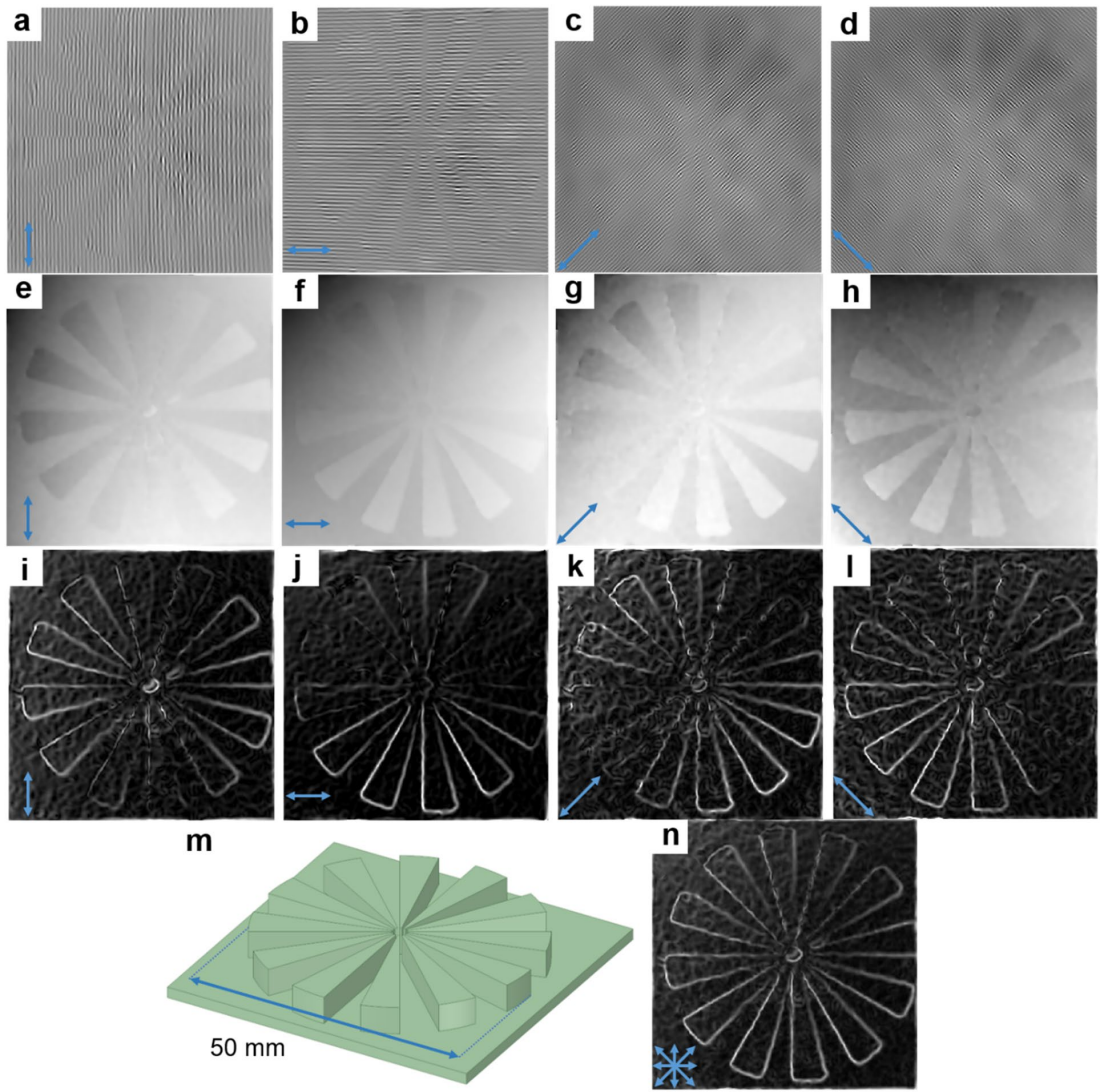
Evaluation of the sensitivity of fringe patterns based on direction. (**a**–**d**) DFPs in the vertical, horizontal, 45°, and 135° directions, respectively. (**e**–**h**) Unwrapped phase maps for DFPs. (**i**–**l**) Detected edge features with Sobel method for maps illustrated in (**e**–**h**), respectively. (**m**) CAD model of the target consisting of twelve spokes spaced 30° apart. (**n**) Result when combining edge features illustrated in (**i**–**l**). The arrows at the bottom left of each panel indicate the direction of the DFP used to reconstruct the corresponding phase map.

In order to assess the sensitivity of the fringe patterns with regard to the directional morphological features, we tested the system on a target that contains edges in a multitude of directions. Differential fringe patterns in the vertical, horizontal, 45°, and 135° directions are shown in Fig. 9a through Fig. 9d, respectively. The phase-demodulation results are shown as unwrapped phase-maps and presented for each direction in Fig. 9e through Fig. 9h. The results were then processed by the Sobel edge detection method to extract the sensed edge for each DFP (shown in Fig. 9i,j,k, l). A CAD model of the target is shown in Fig. 9m. The target contained twelve radially directed spokes each with a height of 4 mm relative to the background plane. It was found that each DFP was blind to the edges nominally parallel to the direction of the fringe pattern. However, a summation of the sensed edges from the individual DFPs yielded a nearly complete picture of all the expected edges (Fig. 9n).

## Discussion

### Major findings

This is the first report of a multispectral FPP method capable of snapshot operation for 3D measurement. In addition, this is the first study to demonstrate the use of a multispectral filter array and multispectral camera for fringe generation and measurement, respectively. Furthermore, the multispectral dot projection pattern provided a novel technique to extract up to 8 unique fringe patterns that resulted in full sampling from a single snapshot and a single projection point. The dot pattern resulted in 4 pairs of complementary π-shifted fringe patterns that were used to increase image resolution and mitigate object glare. Unlike color camera approaches, the higher spectral selectivity of the multispectral camera and MFA provided access to a greater number of unique color channels, had lower cross-talk between color channels, and offered flexibility in dot projection patterning through MFA customization. Also, unlike a color camera, the multispectral camera used near-infrared bands, which are invisible to human vision.

### Speed

The main advantage of MFPP over other FPP methods was that with each camera exposure one 3D representation of the object was acquired. Therefore, the MFPP system was limited only by technical limits imposed by the camera, such as the maximum frame rate and the minimum exposure time. Our camera had a frame rate of 30 FPS, however, cameras with higher frame rate would work as well, since there were no speed limitations imposed by our single point MFA projector. The camera frame rate controls the sampling frequency. Based on the Nyquist theorem, all object movements with a frequency up to half of the frame rate can be sampled without aliasing. The results in Figs. 7 and 8 were captured at 30 FPS and a Nyquist frequency of 15 FPS. The camera was capable of up to 94 FPS with a Nyquist frequency of 47 FPS. Much higher frame rates (up to several thousand FPS) were possible for small regions of interest. For these experiments, an exposure time of 10 ms was used to capture each frame, thereby freezing objects with motion within very short timescales. These could be objects that were moving at high speed through the field of view. The maximum detectable speed was inversely dependent on the camera exposure time and was directly dependent on the projector magnification. However, 3D profilometry of objects with motion at even faster timescales is possible since our camera had a minimum exposure time of 100 µs. The main limitation was the availability of light from the MFA illuminator to provide sufficient camera exposure at these fast time scales. Flashed xenon and LED MFA light sources could be used to achieve these fast timescales with our camera setup. Compared to the methods that use digital projectors^3–6^, which have limited speed and spectral properties as well as more complex instrumentation, our method encoded and detected multiple fringe patterns simultaneously.

### Accuracy

Accuracy was assessed by the MSE between the measured height and the expected height for a hemisphere target (Fig. 4). We found the accuracy to be very high (< 1%) for areas on the object that were facing the MFPP system with poorer accuracy for surfaces that were oblique to the MFPP system. Even still, the accuracy was within ± 10% for even the most oblique surface features. This was evident from the error map shown in Fig. 4e, but also quantitatively in the summary plot shown in Fig. 4f. The plot reveals that for surfaces within 75° of normal the errors were < 3%. However, for surfaces beyond 75° from normal, the error increased substantially. These results suggest MFPP is very robust for highly sloping surfaces. Furthermore, the use of the horizontal and angled fringe patterns available with the MFPP technique could lead to even higher accuracy. For example, in Fig. 4e, the errors at the top and bottom of the hemisphere appear to be lower compared to the errors on the left and right side of the hemisphere. Since only the vertical fringe patterns were used in these instances, we concluded that horizontal fringe patterns could lead to higher accuracies for the left and right sides of the hemisphere and the angled fringes to higher accuracy for areas in between. Another source of error is that the Fourier phase modulation algorithm works on a global level. This could cause an error to spread throughout the entire phase map because of local issues like an abrupt change in the fringe pattern due to an abrupt discontinuity in the surface (see Fig. 5). In addition, due to the snapshot capability of MFPP, additional cameras and illuminators could be positioned around the object to capture the oblique surface features more accurately without any loss in overall acquisition speed.

### Resolution

The lateral resolution of the MFPP system was estimated using a USAF resolution target consisting of several bars, where the spacing between bars covered the expected resolution. This classical approach to resolution measurement allowed us to use FWHM and line profiles to estimate resolution directly. From these measurements, we estimated the lateral resolution be approximately 1 mm. This estimate was expected since the projected fringe period was 2 mm and lateral resolution is known to relate to half of the fringe period^40^. Axial resolution was estimated using a series of hemispherical targets of decreasing diameter. Although this resolution target is not standardized in the literature, it has the advantage of presenting a wide variety of oblique surfaces to the MFPP system for a range of target sizes. From our measurements, we estimated the axial resolution to be approximately 1 mm. As described for the lateral resolution, this estimate was expected due to the 2 mm fringe period. According to the MFPP system, half of the fringe period was equal to the dot size and the dot size was dependent on the magnification lens used in the illuminator and the lens pupil-object distance. Based on Fig. 5e, only 10 pixels were enough to cover a single dot. Therefore, the system should be capable of reaching sub-mm-scale resolutions by decreasing the illuminator magnification, decreasing the lens pupil-object distance (to produce smaller dots), and increasing the camera zoom (to allocate enough pixels to a dot). For large objects, the upper limit of measurements will be dependent on the phase-demodulation and phase unwrapping algorithms. For instance, for phase-demodulation algorithms that use a single wrapped phase map to calculate the absolute unwrapped phase map, such as the FT method, any sudden changes in height that are larger than the fringe size will lead to phase ambiguity.

### MFPP performance unaffected by object surface topography

In traditional FPP systems where a single fringe pattern in a specific direction is used, object topography affects the accuracy of the phase measurements. For example, when the fringe pattern is parallel to a feature with an edge, the feature may not be detected; however, a fringe perpendicular to the same edge will result in the detection of the feature. Furthermore, features with large slopes relative to the optical axis of the illuminator and the camera result in high-frequency fringe patterns that may be under-sampled by the camera^41^. In principle, MFPP can provide fringes in four different directions from a single snapshot, which greatly improves the chances that at least one fringe set will provide accurate local phase measurements (refer to Fig. 9).

### MFPP mitigated degrading effects of glare

Directional illumination of reflective surfaces tends to generate unavoidable glare in the acquired images. Glare can interfere with the fringe patterns, e.g. deformation of the original fringe shapes. Therefore, phase-demodulation algorithms, such as phase shifting and transform-based algorithms, can easily confuse glare as topographical features which increase error. However, by having complementary fringe patterns that have been captured at the same time and the same location, the glare is identical in both frames, but the fringe patterns are phase shifted. This ensures that glare will be diminished by subtracting the two patterns and the corresponding errors will be decreased. Figure 3b,c show the effect of the glare in the retrieved phase maps while the DFP is free from those glare related errors.

### MFPP has greater robustness to under sampling

The MFPP system uses a multispectral camera similar to work by Zhang et al.^18^, but our system required only one projector with a single aperture. The approach is simpler to implement since only one light source was required. Furthermore, projection of the multispectral pattern from a single point greatly simplified triangulation between the projector, sensor, and object surface and resulted in robust phase-to-height conversion. Moreover, the methods that generate a grid pattern and extract orthogonal fringe patterns^3,4,11^ are comparable to only one of the captured spectral bands whereas our method produces four. By having only one grid pattern, the gaps between two adjacent dots will be estimated by the FT filtering which can easily lead to incorrect fringe patterns and under-sampling. Alternatively, our method combines unique filter bands to generate complementary MFPs with higher dot density and thereby improving fringe quality.

### Limitations of this study

Our implementation of MFPP used a single aperture projector and one multispectral camera separated by a well-defined angle with respect to the object. The setup inevitably produced shadows for some regions of objects due to the separation angle. The shadows resulted in phase ambiguities and poorer depth and height estimates in those regions. One potential avenue for improvement is to use multiple complementary projector/camera pairs so that shadows cast by the object for one projector receive illumination from a second projector. Using unique combinations of multispectral fringe patterns for each projector/camera pair would enable simultaneous operation without interference. Other limitations of the MFA projector are the fixed arrangement of the spectral dot pattern, balancing light output at each wavelength, and light throughput. The MFA has narrow spectral transmission characteristics at each band, which requires a high-power light source to sufficiently expose the camera. In our experiment, we used a 100 W QTH source to generate the desired pattern at a distance of 60 cm. Larger distances could be reached by replacing the QTH source with a high-power pulsed LED.

### Implications for the Future

With the development and rapid growth of applications for RGB-D cameras, that can capture both color and depth, MFPP could provide an alternative that can be implemented with simple components. Moreover, utilizing a simple projected light pattern instead of sequenced digital projections offers opportunities to significantly decrease the size of the setup. The MFPP system is suitable for miniaturization, which could enable its use in space-constrained applications such as robotic surgery and mobile device applications such as color 3D video capture.

## Method

### Software

**Figure 10:**
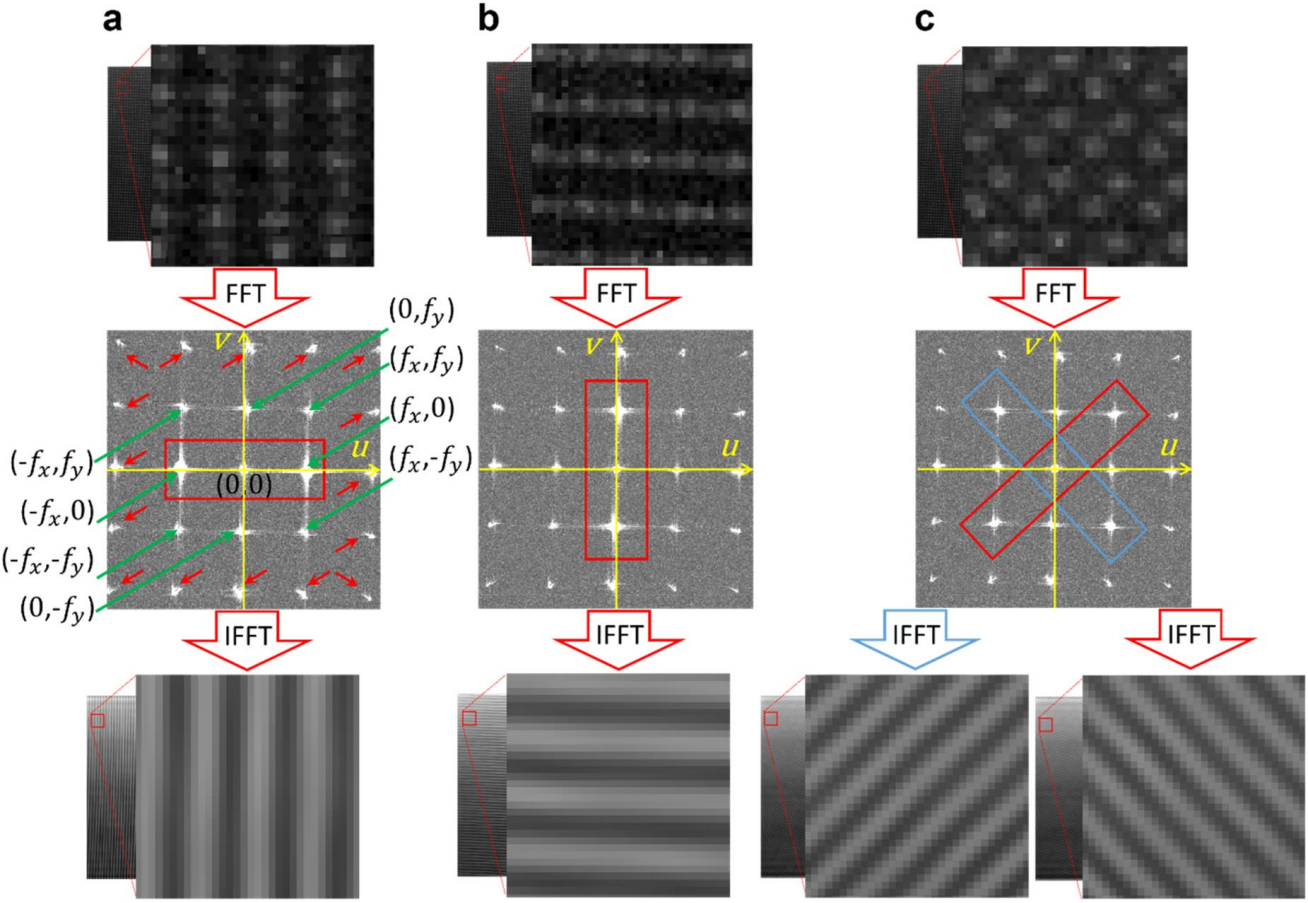
Fringe extraction method. (**a**) From top to bottom: image of the combination of spectral images F1 and F3 showing a magnified view of a small region indicated by the red box; 2D fast Fourier transform (FFT) of data in upper panel showing ± 1 order frequency components (green arrows), and harmonics (red arrows); 2D inverse fast Fourier transform (IFFT) of data in upper panel that has been masked to only include the $\left( {f_{x} ,0} \right)$, $\left( { - f_{x} ,0} \right)$, and $\left( {0,0} \right)$ frequency components. (**b**) Extraction of horizontal fringes from the F1 to F2 combination and (**c**) extraction of two diagonal fringes from the F2 to F3 combination. A magnified view of a small region of the larger data set is indicated by the red box.

Computations and figure creation (Figs. 2, 3, 4, 5, 6, 7, 8, 9, and 10) were performed in MATLAB (Version: 2020b; https://www.mathworks.com; MathWorks, Natick, MA, USA). Some 3D renderings (Figs. 1a,c, and 7a) were created in Microsoft Power Point (Version: 365; https://www.microsoft.com; Microsoft Corporation, Redmond, WA, USA). One 3D rendering (Fig. 1a) was created in Ansys SpaceClaim (Version: 19.1; https://www.ansys.com; Canonsburg, PA, USA).

### Fringe decomposition

The combination of a pair of spectral dot patterns will generate vertical, horizontal, 45°, and 135° MFPs relative to the x-axis. Thus, the four spectral patterns provide six combinations. To extract continuous fringes out of these disjointed MFPs, we applied a fringe extraction method based on Fourier transform^11^. It should be noted that the fringe extraction method does not contribute to the FP phase-demodulation procedure and is only used to illustrate the mathematics of the proposed method. The 2D Fourier transform of an image provides the frequency spectrum containing all spatial frequency components of the image. For an ideal 2D sinusoid fringe pattern, the frequency spectrum expresses three fundamental components for + 1, − 1, and 0 order terms, which indicate the spatial frequency of the intensity variations, its conjugate, and the low variation in background intensity, respectively. For a non-ideal sinusoidal fringe pattern, the Fourier spectrum shows additional terms indicating other frequency orders, e.g., harmonics, and artifacts. Applying inverse Fourier transform over the selected ± 1 and 0 order terms gives an estimate of the ideal sinusoidal fringe pattern. Figure 10 illustrates the sinusoidal fringe pattern decomposition method. Each direction associates with distinct frequency terms in the Fourier spectrum. For example, the combination of F1 and F3 yields a vertical MFP relative to the x-axis (see Fig. 1c), which results in two fundamental spatial frequency components that are symmetrical about the y-axis. The dot-shape of the MFPs causes some spurious frequency components in the Fourier domain. Therefore, relevant terms that need to be selected are $\left( {f_{x} ,0} \right)$, $\left( { - f_{x} ,0} \right)$, and $\left( {0,0} \right)$, where $f_{x}$ is the spatial frequency of the MFP in x-direction also called carrier frequencies in x (Fig. 10a). Figure 10b demonstrates the filtering for horizontal fringes with the relevant frequency terms of $\left( {0,f_{y} } \right)$, $\left( {0, - f_{y} } \right)$, and (0,0) where $f_{y}$ is the spatial frequency (or carrier frequencies) in the y-direction. The diagonal MFPs provided five fundamental components at $\left( {f_{x} ,f_{y} } \right),\left( {f_{x} , - f_{y} } \right),\left( { - f_{x} ,f_{y} } \right),\left( { - f_{x} , - f_{y} } \right)$ and (0,0). In this case, it was possible to select two different sets of frequencies to extract the fringes directed at 45 degrees and 135 degrees (Fig. 10c). By doing so, all six combinations generated eight monochrome sinusoidal fringes.

The mathematical representation of a captured image of a sinusoidal fringe pattern projected on an object can be described as:

1 $$I\left( {x,y} \right) = a\left( {x,y} \right) + b\left( {x,y} \right)\cos \left[ {2\pi \left( {f_{x} x + f_{y} y} \right) + \varphi \left( {x,y} \right)} \right]$$

where *I*, *a*, and *b* denote the fringe pattern, background, and amplitude modulation, respectively, and $\left( {x,y} \right)$ is the pixel coordinate. The argument of the cosine function contains two parts. The first term represents the initial phase of the fringes, shown as $2\pi \left( {f_{x} x + f_{y} y} \right)$. The second term is the phase distribution caused by the surface morphology, shown as *φ(x, y)*.

Equation (1) represents the eight fringe patterns discussed earlier. Since all fringe patterns are derived from the same system with the same light source and at the same time, the background variation can be considered identical for all patterns. However, the individual patterns may have different modulation depths due to the variation in response of the camera at each band. The major differences between these eight fringe patterns is the carrier frequency and the phase. Also, for the complementary fringe patterns, the carrier frequencies are equal, but their phase have a difference of π radians.

### Background and noise-reduced fringe generation

For each direction, the complementary fringe patterns can be considered as double frame two-step phase shifted fringe patterns with a phase step equal to π. In the current work, we utilize these two fringe patterns as independent patterns to generate the DFP. As an example, the vertical complementary fringe patterns produce a DFP as:

2 $$I_{DFP - Vertical} = I_{F1 + F3} - I_{F2 + F4} = \left( {b_{13} + b_{24} } \right) \times \cos \left( {2\pi f_{x} x + \varphi } \right)$$

where $I_{DFP - Vertical}$ is the DFP intensity map, $I_{F1 + F3}$ and $I_{F2 + F4}$ are the extracted complementary fringe patterns such that the indices show the combination of the multispectral frames. Figure 1c shows schematically different combinations of the multispectral frames and Fig. 2 demonstrates the complementary and the corresponding DFPs for all four directions.

### Fourier transform phase demodulation

Fourier transform algorithm is a method of phase-demodulation, which is based on the spatial phase stepping information of the fringe pattern^42,43^. The algorithm follows three steps. *Step 1* Apply FT on the pattern:

Rewriting Eq. (1), using Euler’s rule provides:

3 $$I\left( {x,y} \right) = a\left( {x,y} \right) + c\left( {x,y} \right)e^{{j\left( {2\pi \left( {f_{x} x + f_{y} y} \right)} \right)}} + c^{ * } \left( {x,y} \right)e^{{ - j\left( {2\pi \left( {f_{x} x + f_{y} y} \right)} \right)}}$$

where $c\left( {x,y} \right) = \left( {{1 \mathord{\left/ {\vphantom {1 2}} \right. \kern-\nulldelimiterspace} 2}} \right)b\left( {x,y} \right)e^{{j\varphi_{O} \left( {x,y} \right)}}$ which encompasses the phase information and * denotes the complex conjugate. By taking the FT of this equation we obtain:

4 $${\text{F}}\left\{ {I\left( {x,y} \right)} \right\} = A\left( {u,v} \right) + C\left( {u - f_{x} ,v - f_{y} } \right) + C^{*} \left( {u + f_{x} ,v + f_{y} } \right)$$

where $\left( {u,v} \right)$ represents the spatial frequencies, ${\text{F}}\left\{ \cdot \right\}$ is 2D Fourier transform, and capital letters are the Fourier transform of the corresponding small letters. The three components in Eq. (4) will be well separated in the Fourier domain when the carrier frequencies are higher than the maximum spatial frequencies of the object.

*Step 2* Filter $C$ or $C^{*}$ to extract the object phase distribution, transfer the filtered component to the origin of the Fourier domain to eliminate the carrier frequency, and apply the inverse Fourier transform to get the analytical signal:

5 $${\text{iF}}\left\{ {C\left( {u,v} \right)} \right\} = c\left( {x,y} \right) = \left( {{1 \mathord{\left/ {\vphantom {1 2}} \right. \kern-\nulldelimiterspace} 2}} \right)b\left( {x,y} \right)e^{i\varphi }$$

where ${\text{iF}}\left\{ \cdot \right\}$ is 2D inverse FT function.

*Step 3* Extract the mod 2π wrapped phase by finding the angle of the signal obtained from step 2 and unwrapping the phase to get the absolute phase map.

### Phase-to-height conversion

The phase-to-height conversion is a systematic calibration procedure to extract the surface morphology based on the object phase distribution. Mapping the phase distribution to the surface height can be considered as linear or non-linear operations. Many parameters affect this mapping, including the distance between camera and projector, the orientation of the object/reference planes toward the camera sensor, camera and projector lens distortion, and the diverging nature of the projected fringes. Jia et al.^44^ compared linear and non-linear mapping procedures and concluded that the linear procedure would be more appropriate for objects with shallow depths. On the other hand, nonlinear mapping has the same performance for objects with a small range of heights and is more accurate for objects with a greater range of heights. We have selected the nonlinear approach to convert the phase distribution to a height distribution. This requires measuring the phase distribution of a reference plane located at known distances from the camera. We have used the same procedure for phase estimation as described in Jia et al. (2007) with 20 steps in 2 mm distance increments.

### Post-processing

In some cases, phase maps were multiplied with a binary mask to remove background artifacts. The masks were obtained by thresholding the sum of all four images captured in different spectral bands.
